# Circadian Blood Pressure Profile in Pediatric Patients with Primary Hypertension

**DOI:** 10.3390/jcm11185325

**Published:** 2022-09-10

**Authors:** Michał Szyszka, Piotr Skrzypczyk, Anna Ofiara, Anna Maria Wabik, Radosław Pietrzak, Bożena Werner, Małgorzata Pańczyk-Tomaszewska

**Affiliations:** 1 Department of Pediatrics and Nephrology, Doctoral School, Medical University of Warsaw, 02-091 Warsaw, Poland; 2 Department of Pediatrics and Nephrology, Medical University of Warsaw, 02-091 Warsaw, Poland; 3 Department of Pediatric Cardiology and General Pediatrics, Medical University of Warsaw, 02-091 Warsaw, Poland

**Keywords:** blood pressure dipping, primary hypertension, children, hypertension-mediated organ damage, left ventricular mass, arterial stiffness, cardiovascular risk

## Abstract

Our study aimed to evaluate factors affecting circadian BP profile and its association with hypertension-mediated organ damage (HMOD) in pediatric patients with primary hypertension (PH). The study included 112 children (14.7 ± 2.1 age, 79 boys, 33 girls) with untreated PH. Non-dipping was defined as a nocturnal drop in systolic or diastolic BP (SBP, DBP) < 10%, and a nocturnal drop >20% was defined as extreme dipping. The nocturnal SBP drop was 10.9 ± 5.9 (%), and the DBP drop was 16.2 ± 8.5 (%). Non-dipping was found in 50 (44.6%) children and extreme dipping in 29 (25.9%) patients. The nocturnal SBP decrease correlated with BMI Z-score (r = −0.242, *p* = 0.010) and left ventricular mass index (LVMI) (r = −0.395, *p* = 0.006); diastolic DBP decrease correlated with augmentation index (AIx75HR) (r = 0.367, *p* = 0.003). Patients with a disturbed blood pressure profile had the highest LVMI (*p* = 0.049), while extreme dippers had the highest augmentation index (AIx75HR) (*p* = 0.027). Elevated systolic and diastolic BP dipping were risk factors for positive AIx75HR (OR 1.122 95CI (1.009–1.249) and OR 1.095 95CI (1.017–1.177). We concluded that disturbed circadian BP profile was common in children with PH and should not be considered a marker of secondary hypertension. A disturbed circadian BP profile may be associated with higher body weight. In pediatric patients with PH, non-dipping is associated with increased left ventricular mass, and extreme dipping may be a risk factor for increased arterial stiffness.

## 1. Introduction

Twenty-four-hour ambulatory blood pressure monitoring (ABPM) is currently a more and more easily accessible tool to assess arterial blood pressure (BP) values. ABPM not only helps to detect white coat hypertension and is more useful to control the effectiveness of antihypertensive therapy than office measurements but also provides a valuable insight into the BP variability and fluctuations over the whole day sleep–wake cycle [1]. Moreover, ABPM independence from the operator and available pediatric reference values makes it a useful non-invasive tool to measure cardiovascular risk in children. Up to now, there have been no better methods of BP assessment during the night. Blood pressure values differ during the day and the night. A physiological decline in BP at night is considered a normal circadian pattern—patients with such a nocturnal profile usually are called “dippers”. Patients with an insufficient night fall of BP values are referred to as “non-dippers”. However, more precisely, we can distinguish four different circadian BP rhythms: extreme dippers (over 20% decline in night BP values compared to daytime), dippers (between 20% and 10%), non-dippers (between 10% and 0%), and reverse dippers (nighttime surge in BP) (Table 1) [2]. 

There has been more than three decades of research on circadian BP patterns among hypertensive patients; and yet, there are still conflicting results as to whether dipping status is a valuable predictor of cardiovascular disease (CVD) morbidity and mortality and if it is associated with hypertension-mediated organ damage (HMOD). Studies in adults showed that both a lack of BP decline and excessive drop of BP at night might be indicators of CVD events [3,4]. For example, extreme dippers were found to be at the highest risk of stroke among all dipping patterns [5]. On the other hand, a recent meta-analysis states that only reverse dippers are at high risk of complications [6]. Similar discrepancies exist regarding whether normalizing blood pressure profile might improve cardiovascular risk. Of note, the results from a recently published yet strongly criticized Hygia study showed that prescribing one of the antihypertensive drugs at bedtime can improve dipping values and diminish the occurrence of CVD events [7]. There are also some scarce and conflicted data in children [8,9]. Some papers suggest that non-dippers are associated with endothelial dysfunction and thicker intima-media [10]. On the other hand, Seeman et al. found no difference in HMOD assessed as a left ventricular mass index between dippers and non-dippers [11]. 

Therefore, our study aimed to assess dipping status, its determinants, and its relationship with precisely evaluated HMOD: left ventricular mass, urinary albumin excretion, and indicators of arterial damage: common carotid artery intima-media thickness (cIMT), arterial stiffness (augmentation index—AIx75HR, and aortic pulse wave velocity—aPWV) in a cohort of pediatric patients with untreated primary hypertension (PH).

## 2. Materials and Methods

### 2.1. Study Group

The sample size estimated based on the available literature with a statistical power of 0.8, *p* = 0.05, and an effect size of 0.50 should be at least 64 (effect size calculated for the presence of any HMOD) [6,7,10,11]. The study included 112 children with newly diagnosed, untreated PH recruited among patients hospitalized in one pediatric tertiary nephrology center between December 2017 and February 2021. The youngest patient was 5.5 years old and the oldest 17.92 years old. The inclusion criterion was arterial hypertension diagnosed according to the European Society of Hypertension guidelines from 2016 [12] and confirmed by ABPM [1]. The exclusion criteria were: white-coat hypertension (excluded by ABPM) [1], secondary hypertension, pharmacological and/or non-pharmacological treatment of hypertension, confirmed or suspected heart, renal, vascular, or endocrine pathology, chronic inflammatory diseases (e.g., inflammatory bowel disease), and acute inflammatory infections (temporary exclusion for a time of 4 weeks). The study was characterized as an observational, cross-sectional one. 

### 2.2. Ethical Issues

Before the initiation of the study, approval from the local bioethics committee was obtained (document no. KB/58/2016, 15 March 2016). All procedures were performed in accordance with the Declaration of Helsinki on the treatment of human subjects and its later amendments. All legal representatives of the patients, and patients if aged 16 years and older, signed informed consent to participate in the study.

### 2.3. Clinical Parameters

All examinations were performed upon admission using the same protocol. The following clinical parameters were assessed: age (years), sex, duration of arterial hypertension (months), duration of pregnancy (weeks), birth weight (g), and basic anthropometric parameters such as height (cm), weight (kg), and calculated body mass index (BMI) (kg/m^2^). If possible, all measurements were compared with Polish normative data and expressed as Z-score [13]. Overweightness and obesity were assigned according to World Health Organization definitions: BMI ≥ 85th and <95th percentile, and ≥95th percentile, respectively [14].

### 2.4. Laboratory Tests

Laboratory tests were conducted using standard local laboratory methods. The blood was drawn after at least 8 h of fasting in a sitting position in the morning (between 7:00 and 9:00 a.m.). The following complete biochemical tests were assessed: serum potassium (mmol/L), sodium (mmol/L), creatinine (mg/dL), uric acid (mg/dL), total, low-density lipoprotein (LDL), high-density lipoprotein (HDL) cholesterol (mg/dL), and triglycerides (mg/dL). Lipid disturbances were classified following [15], and hyperuricemia was classified as serum uric acid ≥ 5.5 (mg/dL) after Feig et al. [16]. 24-h urine collection was used to assess urinary albumin (mg/L), sodium (mmol/L), and creatinine (mg/dL), and 24-h urinary albumin excretion (UAE) (mg/24 h) and 24-h urinary sodium excretion (mmol/kg/24 h) were calculated; UAE ≥ 30 (mg/24 h) was assumed as an abnormal value [2,12]. Additionally, in 26 (23.2%) subjects, urinary potassium (mmol/L) was evaluated and 24-h urinary potassium excretion was calculated (mmol/kg/24 h). Estimated glomerular filtration rate (eGFR) was determined according to 2009 Schwartz creatinine-based formula (mL/min/1.73 m^2^) [17]. All tests were conducted as early as possible after collection, within a maximum of 2 h, without previous freezing. 

### 2.5. Blood Pressure Measurements and Dipping Status

The BP measurements were carried out according to protocols suitable for pediatric populations [12] and described in detail in our previous manuscripts [18,19]. Briefly, peripheral office BP was assessed oscillometrically by Welch Allyn VSM Patient Monitor 300 (Welch Allyn Inc., Skaneateles Falls, NY, USA) (mmHg) and expressed as Z-scores using Polish normative data [20]. Suntech Oscar 2 oscillometric device (SunTech Medical, Inc., Morrisville, NC, USA) was used to evaluate 24-h blood pressure parameters and circadian blood pressure profile, and data were interpreted according to current pediatric recommendations [1]. Systolic and diastolic blood pressure dipping was calculated as a difference between mean daytime blood pressure and mean nighttime blood pressure expressed as a percentage of the daytime value. Impaired night pressure drop (non-dipping pattern) was defined as a night drop of systolic or diastolic pressure below 10%, and a night drop in systolic or diastolic pressure above 20% was defined as extreme dipping (Table 1). To sum up, the following ABPM parameters were included in the final analysis: systolic, diastolic, and mean arterial blood pressure (SBP, DBP, MAP, respectively) during activity and resting periods, and during 24 h (mm Hg), 24 h MAP Z-score, heart rate (beats per minute), and nocturnal blood pressure dipping (%).

### 2.6. Echocardiography and assessment of Properties of Arteries

Left ventricle mass index (LVMI) was calculated with parameters drawn from echocardiography (ECHO) measurements using M-mode assessment of the left ventricle with simultaneous recording of ECG in the second limb lead. The detailed methodology was described in our recently published manuscript [21]. Measurements were carried out with Philips iE33 device, using an S5-1 transducer (Philips, Amsterdam, the Netherlands). The following parameters were collected in the end-diastolic phase: the interventricular septum transverse diameter (IVSDd) (mm), left ventricular diastolic diameter (LVDd) (mm), and left ventricular posterior wall diameter (LVPWd) (mm)). Left ventricular mass (LVM, (g)) was calculated from the Deveraux equation and indexed according to the DeSimone formula to normalize the results to body size (g/m^2.7^) [22]. Left ventricular hypertrophy (LVH) was defined as LVMI ≥ 95c. for age and sex in accordance with normal values used in the pediatric population [23]. 

Assessment of arterial damage and central properties of the arteries were also described thoroughly in manuscripts of our previous studies [24,25]. SphygmoCor (AtCor Medical Pty Ltd., Sydney, Australia) and applanation tonometry technique were used to assess central blood pressure, arterial pulse waveform parameters from the radial artery, and aortic (carotid–femoral) pulse wave velocity (aPWV). The parameters included in the analysis were: aortic (central) office systolic, diastolic, and mean blood pressure (AoSBP, AoDBP, AoMAP (mm Hg)), augmentation pressure (AP) (mm Hg), augmentation index (AIx (%)), as well as AIx normalized to the heart rate of 75 beats per minute (AIx75HR (%)), and subendocardial viability ratio (SEVR—Buckberg index) (%). aPWV was presented as (m/s) and (Z-score) based on available normative pediatric data for applanation tonometry devices [26]. 

Additionally, left and right common carotid artery intima-media thickness (cIMT) was evaluated with a 13-MHz linear transducer (Aloka Prosound Alpha 6, Hitachi Aloka Medical, Mitaka, Japan), using already described methods [24] and expressed both in (mm) and as Z-score [27].

aPWV ≥ 95th percentile, cIMT ≥ 95th percentile, and a positive value of AIx75HR were assumed as indicators of arterial damage in the studied patients. 

### 2.7. Statistical Analysis

Collected data were archived in an anonymized form (Excel 365, Microsoft 365, Microsoft, Redmond, WA, USA) with password-protected user-level access. Statistical analysis was conducted using Dell Statistica 13.0 PL software (TIBCO Software Inc., Palo Alto, CA, USA). First, the Shapiro–Wilk test was used to assess the normality of data distribution. Continuous variables were presented as mean ± standard deviation (SD) and interquartile range (IQ). The following tests were used during statistical analysis: student T-test, Mann-Whitney U test, ANOVA, Kruskal–Wallis ANOVA, Spearman’s rank correlation, Pearson correlation, chi-squared test, Fisher’s exact test, receiver operating characteristics (ROC) analysis, and logistic regression. In the logistic regression model, unadjusted odd ratios for the presence of HMOD and blood pressure dipping as continuous predictors were calculated. We did not include blood pressure values in the model as there were no significant correlations between 24 h blood pressure (SBP, DBP, MAP, MAP Z-score) and blood pressure dipping. Test results with a *p*-value < 0.05 was considered of statistical significance.

## 3. Results

### 3.1. Clinical Characteristics and Biochemical Parameters

Basic clinical parameters and results of laboratory tests of all examined patients were depicted in Table 2. In the studied cohort, 71% of the patients were boys. The mean duration of hypertension was 14.6 months, and the majority of the patients were born at term (80/112—71%). Only 31% (35/112) of all examined children with hypertension had normal BMI; most of them were either overweight (46/112—41%) or obese (31/112—28%). Hyperuricemia was revealed in 62/112 (55.4%) of the patients. Any form of lipid disturbances, according to [15], was found in 75/112 (67.0%) patients. A figure of 18/112 (16.1%) patients had abnormal urinary albumin excretion. 

### 3.2. Blood Pressure and Markers of Hypertension-Mediated Organ Damage

**Figure 1:**
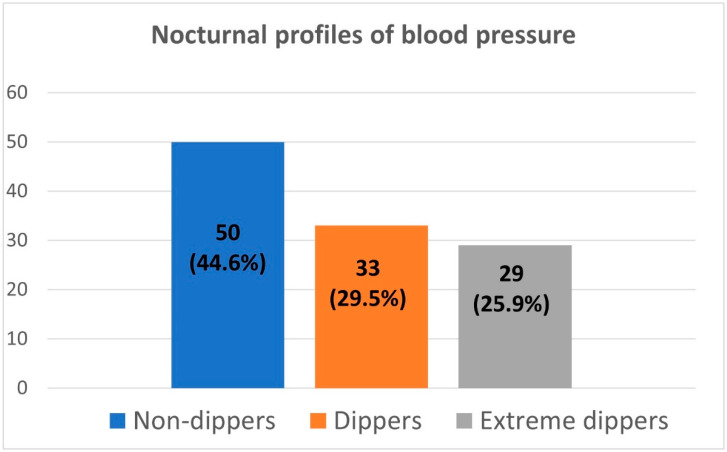
Dipping patterns distribution among studied population (numbers of patients).

Office peripheral and central blood pressure and ambulatory blood pressure are depicted in Table 3. Based on ABPM results, 78/112 (70%) patients had isolated systolic hypertension, 33/112 (29%) subjects had systolic–diastolic hypertension, and 1/112 (1%) obese boy aged 13.7 had isolated diastolic hypertension. The mean systolic blood pressure dipping in ABPM was 10.9 ± 5.9 (%), and the mean diastolic blood pressure dipping was 16.2 ± 8.5 (%). When divided upon dipping status, almost half of the patients were classified as non-dippers and more than one-fourth as extreme dippers. The remaining subjects had normal dipping status. There were no reverse dippers in our study group (Figure 1). The non-dipping phenomenon was due to a lack of SBP dipping in 48/50 (96%) cases and in 21/50 (42%) due to DBP non-dipping.

The parameters of hypertension-mediated organ damage were presented in Table 4. Left ventricular hypertrophy was revealed in 26/112 (23%) patients, abnormal aPWV in 5/112 (4%), abnormal AIx75HR in 24/112 (21%), abnormal cIMT in 22/112 (20%), and abnormal UAE in 18/112 (16%) subjects. At least one form of HMOD was found in 68/112 (61%) of all the patients. 

### 3.3. Determinants of BP Dipping and Association between BP Dipping and HMOD

The significant correlations of systolic and diastolic BP dipping with clinical and laboratory parameters in the whole group of 112 children are shown in Table 5. There was a significant negative correlation between systolic blood pressure dipping and body weight as well as BMI; both expressed as Z-scores. In 26/112 patients where data were available, urinary potassium excretion correlated positively with both systolic and diastolic BP dipping. No other significant correlations were revealed between clinical and biochemical data and BP dipping. 

The correlations of central systolic blood pressure and markers of HMOD with BP dipping are depicted in Table 6. LVMI correlated negatively with systolic blood pressure dipping, and the augmentation index (AIx75HR) correlated positively with diastolic blood pressure dipping. There were no other significant correlations concerning dipping status and HMOD in the studied group of patients. 

All children were divided into three groups according to their BP dipping pattern—dippers, non-dippers, and extreme dippers. Results of differences in studied parameters between the groups are depicted in Table 7. Statistical analysis showed no significant difference between these three groups in terms of analyzed clinical and biochemical parameters except for urinary potassium excretion, but the latter analysis was limited to 26 patients. The groups also did not differ significantly in office peripheral and central blood pressure, and 24-h ambulatory blood pressure. The analysis of HMOD revealed no significant differences between the groups in terms of urinary albumin excretion (*p* = 0.083), aortic pulse wave velocity (*p* = 0.213 and *p* = 0.527), and common carotid artery intima-media thickness (*p* = 0.484 and *p* = 0.357). The left ventricular mass index was the highest in the non-dipper group, significantly higher compared to the dipper group (*p* = 0.049), and the augmentation index was the highest in the extreme dipper group, significantly higher compared to the non-dipper group (*p* = 0.027).

Analysis by logistic regression revealed that elevation in systolic and diastolic BP dipping increases the risk for abnormal AIx75HR (odds ratio (OR) 1.122, 95 confidence interval (95CI) (1.009–1.249), and OR 1.095, 95CI (1.017–1.177), respectively) (Table 8). 

ROC analysis demonstrated good diagnostic profiles for the presence of abnormal AIx75HR. The cut-off value of systolic and diastolic BP dipping to predict positive AIx75HR was 7.4 (AUC 0.650, 95CI (0.512–0.789), *p* = 0.034) and 15.7 (AUC 0.706, 95CI (0.576–0.835), *p* = 0.002). 24-h mean arterial pressure Z-score and other indices of 24-h blood pressure did not predict AIx75HR with statistical significance (AIx75HR (%) vs. 24 h MAP Z-score: AUC 0.506, 95CI (0.358–0.654), *p* = 0.939).

## 4. Discussion

Our single-center cross-sectional study aimed to assess the dipping status in pediatric patients with untreated primary (essential) hypertension. First, we revealed that the non-dipping phenomenon is present in as many as half of the patients, and almost a quarter of them can be classified as extreme dippers leaving only a minority of patients with normal dipping status. Secondly, body mass and body mass index were significant determinants of dipping status. As for the analysis of the relation between dipping status and HMOD, systolic blood pressure dipping correlated negatively with left ventricular mass index, and non-dippers were found to have significantly higher left ventricular mass compared to dippers. Conversely, the augmentation index correlated positively with blood pressure dipping, and extreme dippers were characterized by the highest augmentation index. The latter association was confirmed by logistic regression and ROC analysis. Interestingly, no significant associations were revealed between central blood pressure, aortic pulse wave velocity, common carotid artery intima-media thickness, and dipping status.

We analyzed patients at one tertiary nephrology center with confirmed primary hypertension. We managed to collect a group of more than 100 treatment-naive patients. Our cohort can be considered representative of adolescent patients with primary hypertension. The mean age in our group was between 14.5 and 15 years, and the male gender was predominant. Additionally, more than 2/3 of the patients were overweight and obese—similar percentages are found in studies by other authors [28,29,30,31]. We found hyperuricemia in a large portion of patients, a hallmark of primary hypertension, and uric acid may be involved in its pathogenesis [16]. Noteworthy, the predominant vascular phenotype in our cohort was isolated systolic hypertension, which also coincides with the results of other authors’ works [32].

Arterial hypertension is the most important independent risk factor for cardiovascular morbidity and mortality worldwide. A particularly strong relationship is found between systolic blood pressure and the risk of stroke [2]. Hard end-points are fortunately virtually non-existent in pediatrics, and incident cardiovascular events are observed primarily in patients with vasculopathies and additional risk factors such as chronic kidney disease, vasculitis, familial hypercholesterolemia, or recently as a consequence of COVID-19 infection [33,34,35]. In contrast, subclinical hypertension-mediated organ damage (HMOD) is common in adolescents with PH. Evaluation of HMOD is of particular importance because it indicates the need for antihypertensive treatment, on the one hand, and, on the other, it identifies patients who should be subjected to special surveillance. According to ESH recommendations, assessment of left ventricular mass by ECHO is the most important, objective way to assess HMOD [12]. Additional methods include assessment of urinary albumin excretion and evaluation of arterial lesions: especially cIMT and central arterial stiffness assessed as aortic PWV and additionally, AIx75HR. In our cohort, any form of HMOD was observed in about half of the patients, which also coincides with the findings of other authors [36,37,38].

For many years, the importance of assessing not only the BP value itself but also other derivatives such as pulse pressure or BP variability has been raised. Assessment of pressure variability can be done in the long-term (visit-to-visit) range but also in the shorter term. Since the introduction of the ABPM study into widespread use, circadian variability has become the most commonly assessed variability. Arbitrarily years ago, it was accepted that the normal nocturnal drop in blood pressure was 10% (dippers vs. non-dippers). In subsequent years, data emerged indicating that it was also useful to separate patients with a high (>20%) nocturnal drop (extreme dippers) and those whose nighttime pressure increases (reverse dippers) [1,2]. Risk factors for impaired nocturnal BP drop have been identified. It has been repeatedly shown that secondary forms of AH (renal; endocrine e.g., primary hyperaldosteronism, pheochromocytoma/paraganglioma; autonomic dysfunction) are characterized by a higher prevalence of an impaired blood pressure profile [39]. As for pediatric data, Flynn’s classic paper found that patients with secondary AH were characterized by higher nighttime diastolic pressure and an impaired diurnal profile of diastolic pressure. It is worth noting that Flynn based his conclusions on a small group of patients [40]. In our study published in the local press, we compared 31 patients with PH and 33 patients with renal hypertension. Patients with PH were characterized by a tendency toward a lower diastolic pressure load at night and a greater decrease in systolic and diastolic blood pressure [41]. Interestingly, in our cohort of pediatric patients with PH, as many as about half of the patients with confirmed PH were characterized by non-dipping and almost a quarter by extreme dipping. This indicates that assessment of the diurnal profile alone should not be an indication to look for or exclude secondary forms of hypertension.

The nocturnal drop in systolic blood pressure correlated negatively with body weight and BMI (both values normalized to pediatric values). These results indicate that overweight and obesity are risk factors for impaired nocturnal pressure drop. Other studies have found similar results in adults and children [42,43]. Obesity and overweight are associated with overactivation of the sympathetic nervous system and hyperinsulinism, which may be responsible for elevated nighttime blood pressure. In addition, patients with excess body weight may suffer from obstructive sleep apnea (OSA), a well-known risk factor for nocturnal hypertension [44].

A sub-analysis of 26 patients showed a strong positive relationship between urinary potassium excretion and a nocturnal blood pressure drop. Evaluation of urinary excretion of the component is a simple marker of its dietary supply. Population studies have shown that increasing dietary potassium is associated with a reduction in blood pressure and cardiovascular risk. Bankir et al. found a positive association between daytime urinary potassium excretion and blood pressure dipping [45]. Conversely, in a study by Libianto et al., no relation between urinary potassium excretion and blood pressure dipping was found in adults with diabetes mellitus [46]. Further studies on larger numbers of pediatric patients evaluating the relationship between dietary sodium and potassium supply and the diurnal pressure profile are needed.

Our study showed that an impaired circadian blood pressure profile is a risk factor for left ventricular hypertrophy. These findings are consistent with the results of numerous adult studies [47]. Nevertheless, there are doubts about the real significance of non-dipping for developing left ventricular hypertrophy. It is known that the importance of diurnal blood pressure profile is significantly lower in treated patients and, in addition, some studies indicate that the value of nighttime and weighted 24-h average BP is the real predictor of left ventricular hypertrophy [48]. Seeman showed no relationship between blood pressure dipping and left ventricular mass or left ventricular hypertrophy in pediatric patients. However, what is noteworthy is that the authors analyzed both patients on antihypertensive treatment (87/114) and patients with various causes of hypertension (80/114 children with renoparenchymal hypertension) [11]. Other factors, such as renal function, may have played a role in the failure to show a relationship between left ventricular mass and dipping status in the cited study. Similarly, a pediatric Chinese study revealed no relation between BP dipping and left ventricular hypertrophy [9]. In this case, the difference in results could be derivative of younger age (13 years), different ethnicity, and different thresholds for LVH.

In addition, we showed that the phenomenon of extreme dipping is associated with an increase in the augmentation index. Patients defined as extreme dippers are characterized by a significant (>20%) decrease in nighttime blood pressure. Palatini, in a study involving more than 10,000 adults, showed that extreme dipping is a risk factor for cardiovascular complications, but only in patients over the age of 70 [4]. Interestingly, recently extreme dippers were found to have an increased risk for left ventricular hypertrophy [49,50]. Our results indicate that excessive nocturnal blood pressure drop might also be associated with adverse arterial changes. Noteworthy, unfortunately, we did not show such a relationship for a parameter that is the gold standard for assessing vascular stiffness—aortic pulse wave velocity. It should also be stressed that some data indicate an opposite association between dipping status and arterial stiffness. Two adult Asian studies [51,52] and one small Portuguese pediatric study [8] showed that augmentation index was positively associated with rather non-dipping not extreme dipping phenomenon.

Elevated urinary albumin excretion indicates subclinical endothelial damage not only in the kidneys and is an independent risk factor for cardiovascular morbidity and mortality [2]. Evaluation of albuminuria is recommended in both adults and children with hypertension [2,12]. We did not demonstrate a statistically significant association of albuminuria with BP dipping in our cohort. Otherwise, some of the data in adults suggest an association of impaired diurnal profile with albuminuria [53], e.g., in patients with uncontrolled nocturnal hypertension [54]. One pediatric study also revealed that a higher urine protein-to-creatinine ratio was associated with significantly higher odds of non-dipping [55]. Further studies, including prospective analyses on the significance of this phenomenon for the development of HMOD and hard-end points, are needed.

To date, two questions remain unresolved: first, whether we can effectively influence the circadian blood pressure profile and whether restoring a normal blood pressure profile reduces cardiovascular risk. Current European guidelines recommend prescribing treatment with long-acting drugs in a single morning dose (in adults, preferably a single pill composition) [2]. Most studies did not show any additional benefit from supplying an antihypertensive drug in the evening. The only study that unequivocally showed a benefit of drug delivery in the evening is the HYGIA study [7]. Nevertheless, after its publication, a debate swept through the literature questioning its credibility. Even more so, there are no such data in children. Here, too, there is a need for interventional studies that could demonstrate the benefit of drug delivery in the evening in pediatric patients with nocturnal hypertension.

The strength of our study is the relatively large group of pediatric patients with untreated (including non-pharmacological treatment) hypertension and the in-depth analysis of HMOD. Limitations are certainly the cross-sectional nature of the study, and the lack of validation of the OSCAR 2 SUNTECH device in the pediatric population (although the device has been validated in adults and has shown utility in pediatric patients in numerous of our publications [18,19,25,41]). The incomplete analysis of urinary potassium excretion is also a limitation. Patients were also not screened for obstructive sleep apnea and sleep quality; the ABPM could have affected the latter test itself.

## 5. Conclusions

Although the nocturnal drop in blood pressure (dipping) is a standard parameter assessed by ABPM, its significance, especially in the pediatric population, is not clear. Our single-center, cross-sectional study showed that both a disturbed circadian blood pressure profile (non-dipping) and a large nocturnal drop (extreme dipping) are common phenomena in pediatric patients with primary hypertension. Excess body weight was associated with disturbed blood pressure dipping. Noteworthy, the impaired circadian blood pressure profile was associated with increased left ventricular mass. On the other hand, extreme dipping was related to increased arterial stiffness. Our study indicates that an impaired circadian blood pressure profile should not be considered a marker of secondary hypertension but rather a marker of risk for hypertension complications. The cross-sectional nature of our study does not answer the question of whether we have therapeutic options to influence the circadian profile of blood pressure and whether its normalization is associated with regression of HMOD. There is no doubt that further prospective studies targeting the issue of the circadian blood pressure profile in adolescent patients with primary hypertension are needed.

## Figures and Tables

**Table 1:** Nocturnal blood pressure dipping patterns according to the European Society of Hypertension [2].

Dipping Pattern	Night Drop in Blood Pressure (Difference between Day and Night Compared to Day)
Extreme dipper	≥20%
Dipper	≥10% but <20%
Non-dipper	≥0% but <10%
Reverse dipper	<0%

**Table 2:** Basic clinical and biochemical parameters in the study group.

Parameter	Value in the Study Group n or Mean ± SD (IQR)
Number of patients (n)	112
Age (years)	14.7 ± 2.1 (13.8–16.8)
Boys / Girls	79/33
BMI Z-score	1.36 ± 0.95 (0.77–2.04)
Duration of hypertension (months)	14.6 ± 21.5 (3–14)
Duration of pregnancy (weeks)	38.6 ± 2.8 (38–40)
Birth weight (g)	3242 ± 636 (2920–3710)
eGFR acc. Schwartz formula (mL/min/1.73 m^2^)	98 ± 20.5 (83.5–113.2)
Uric acid (mg/dL)	5.9 ± 1.3 (5.2–6.8)
Sodium (mmol/L)	142.7 ± 2.0 (141–144)
Potassium (mmol/L)	4.5 ± 0.3 (4.3–4.7)
Total cholesterol (mg/dL)	156.8 ± 25.9 (137–171)
LDL cholesterol (mg/dL)	84.6 ± 22.4 (68–99)
HDL cholesterol (mg/dL)	50.7 ± 13.2 (41–55)
Triglycerides (mg/dL)	107.2 ± 52.3 (67–141)
Urinary albumin excretion (mg/24 h)	34.2 ± 8.1 (5.3–20.7)
Urinary sodium excretion (mmol/kg/24 h)	2.2 ± 1.2 (1.5–3.0)
Urinary potassium excretion (mmol/kg/24 h) *	0.8 ± 0.3 (0.6–0.9)

n—number of patients, SD—standard deviation, IQR—interquartile range, BMI—body mass index, eGFR—estimated glomerular filtration rate, LDL—low-density lipoprotein, HDL—high-density lipoprotein. *—26/112 patients.

**Table 3:** Office peripheral, office central, and peripheral ambulatory blood pressure.

Parameter	Study Group ± SD (IQR)
Peripheral office SBP [mmHg]	133.9 ± 10.8 (126–143)
Peripheral office SBP Z-score	1.68 ± 0.81 (1.06–2.20)
Peripheral office DBP [mmHg]	79.3 ± 11.1 (73–85)
Peripheral office DBP Z-score	2.01 ± 1.46 (1.17–2.48)
Central office SBP [mmHg]	112.0 ± 9.3 (106–118)
Central office DBP [mmHg]	81.0 ± 11.2 (74–87)
Central office MAP [mmHg]	95.9 ± 10.0 (89–101)
24 h ambulatory SBP [mmHg]	133.2 ± 6.8 (129–138)
24 h ambulatory DBP [mmHg]	72.6 ± 6.6 (68–77)
24 h ambulatory MAP [mmHg]	92.7 ± 5.9 (88–97)
24 h ambulatory MAP Z-score	1.6 ± 1.3 (0.9–2.1)
24 h ambulatory HR [bpm]	79.6 ± 11.2 (72–89)
activity ambulatory SBP [mmHg]	137.0 ± 7.3 (132–142)
activity ambulatory DBP [mmHg]	75.8 ± 7.5 (71–80)
activity ambulatory MAP [mmHg]	95.3 ± 10.9 (91–100)
activity ambulatory HR [bpm]	83.4 ± 12.0 (76–93)
resting ambulatory SBP [mmHg]	121.9 ± 8.6 (116–128)
resting ambulatory DBP [mmHg]	63.1 ± 7.1 (59–67)
resting ambulatory MAP [mmHg]	82.7 ± 6.9 (79–87)
resting ambulatory HR [bpm]	68.9 ± 12.0 (62–76)
Systolic DIP [%]	10.9 ± 5.9 (7.3–14.8)
Diastolic DIP [%]	16.2 ± 8.5 (11.6–21.2)

SD—standard deviation, IQR—interquartile range, SBP—systolic blood pressure, DBP—diastolic blood pressure, MAP—mean arterial pressure, HR—heart rate, bpm—beats per minute, DIP—blood pressure dipping.

**Table 4:** Hypertension-mediated organ damage in the studied children.

Parameter	Study Group ± SD (IQR)
ECHO IVSd [mm]	7.6 ± 1.8 (6–9)
ECHO LVPWd [mm]	7.8 ± 1.7 (7–9)
ECHO LVEDd [mm]	48.7 ± 5.3 (46–53)
ECHO LVM [g]	168.3 ± 60.0 (121.6–205.2)
ECHO LVMI [g/m^2.7^]	40.8 ± 11.4 (32.5–47.7)
aPWV [m/s]	5.4 ± 0.9 (4.7–5.8)
aPWV Z-score	−0.01 ± 1.09 (−0.76–0.68)
AP [mm Hg]	−1.6 ± 4.9 (−4.0–0.0)
AIx [%]	−5.4 ± 14.7 (−13.7–1.0)
AIx75HR [%]	−2.9 ± 13.2 (−11.3–4.0)
Buckberg SEVR [%]	165.5 ± 42.3 (134–194)
cIMT [mm]	0.46 ± 0.07 (0.40–0.50)
cIMT Z-score	1.28 ± 1.41 (0.16–2.29)

SD—standard deviation, IQR—interquartile range, ECHO—echocardiography, IVSd—interventricular septum transverse diameter in diastole, LVPWd—left ventricular posterior wall diameter in diastole, LVDd—left ventricular end-diastolic diameter, LVM—left ventricular mass, LVMI—left ventricular mass index, aPWV—aortic pulse wave velocity, AP—augmentation pressure, AIx—augmentation index, AIx75HR—augmentation index normalized to heart rate 75 beats per minute, SEVR—subendocardial viability ratio, cIMT—common carotid artery intima-media thickness.

**Table 5:** Significant correlations of clinical and biochemical parameters with dipping status (continuous variable) in patients with primary hypertension.

Analyzed Parameter	r	*p*
SBP DIP [%] vs. weight Z-score	−0.191	0.043
SBP DIP [%] vs. BMI Z-score	−0.242	0.010
SBP DIP [%] vs. urinary potassium excretion * [mmol/kg/24 h]	0.702	<0.001
DBP DIP [%] vs. urinary potassium excretion * [mmol/kg/24 h]	0.540	0.011

SBP—systolic blood pressure, DBP—diastolic blood pressure, DIP—blood pressure dipping, BMI—body mass index. *—26/112 patients.

**Table 6:** Correlations of the parameters of hypertension-mediated organ damage with blood pressure dipping (continuous variable).

Analyzed Parameter	r	*p*
SBP DIP [%] vs. AoSBP [mm Hg]	−0.144	0.252
DBP DIP [%] vs. AoSBP [mm Hg]	−0.032	0.801
SBP DIP [%] vs. LVMI [g/m^2.7^]	−0.395	0.006
DBP DIP [%] vs. LVMI [g/m^2.7^]	−0.139	0.350
SBP DIP [%] vs. aPWV Z-score	0.071	0.584
DBP DIP [%] vs. aPWV Z-score	0.080	0.537
SBP DIP [%] vs. AIx75HR [%]	0.206	0.108
DBP DIP [%] vs. AIx75HR [%]	0.367	0.003
SBP DIP [%] vs. cIMT Z-score	0.150	0.235
DBP DIP [%] vs. cIMT Z-score	0.169	0.182
SBP DIP vs. urinary albumin excretion [mg/24 h]	0.058	0.573
DBP DIP vs. urinary albumin excretion [mg/24 h]	−0.036	0.724

SBP—systolic blood pressure, DBP—diastolic blood pressure, DIP—blood pressure dipping, AoSBP—central systolic blood pressure, LVMI—left ventricular mass index, aPWV—aortic pulse wave velocity, AIx75HR—augmentation index normalized to heart rate 75 beats per minute, cIMT—common carotid artery intima-media thickness.

**Table 7:** Differences in analyzed parameters between dippers and non-dippers in the studied children (mean ± SD).

Parameter	Non-Dippers	Dippers	Extreme Dippers	*p*
Number of patients	50	33	29	-
Age [years]	14.7 ± 2.5	14.9 ± 2.6	14.6 ± 3.3	0.910
Sex (boys/girls)	32/18	27/6	20/9	0.516
BMI Z-score	1.5 ± 1.1	1.2 ± 0.9	1.3 ± 0.9	0.298
Duration of hypertension [month]	15.6 ± 24.8	16.8 ± 22.8	10.5 ± 12.6	0.531
Duration of pregnancy [weeks]	37.9 ± 3.7	39.5 ± 1.4	38.3 ± 2.2	0.287
Birth weight [g]	3102 ± 676	3328 ± 660	3396 ± 499	0.436
eGFR [mL/min/1.73 m^2^]	99.4 ± 21.1	95.2 ± 17.0	98.7 ± 23.0	0.662
Uric acid [mg/dL]	6.0 ± 1.4	5.9 ± 1.2	5.8 ± 1.3	0.894
Sodium [mmol/L]	142.7 ± 1.9	142.9 ± 1.7	142.4 ± 2.4	0.624
Potassium [mmol/L]	4.5 ± 0.3	4.6 ± 0.4	4.4 ± 0.3	0.149
Total cholesterol [mg/dL]	152.9 ± 22.3	158.9 ± 25.5	161.1 ± 31.7	0.364
LDL cholesterol [mg/dL]	83.6 ± 21.4	81.4 ± 21.4	89.8 ± 25.2	0.360
HDL cholesterol [mg/dL]	48.5 ± 12.6	54.8 ± 15.9	50.2 ± 9.9	0.119
Triglycerides [mg/dL]	107.0 ± 48.8	107.3 ± 61.2	107.3 ± 49.1	1.000
Urinary albumin excretion [mg/24 h]	53.5 ± 11.2	10.4 ± 7.2	31.2 ± 5.9	0.083
Urinary sodium excretion [mmol/kg/24 h]	2.3 ± 1.2	2.2 ± 1.0	2.4 ± 1.3	0.846
Urinary potassium excretion [mmol/kg/24 h] *	0.6 ± 0.2	1.0 ± 0.4	1.2 ± 0.2	<0.001 ^1^
Peripheral office SBP [mmHg]	136.7 ± 10.5	132.3 ± 9.9	131.1 ± 9.9	0.183
Peripheral office SBP Z-score	2.00 ± 0.95	1.44 ± 0.65	1.49 ± 0.53	0.113
Peripheral office DBP [mmHg]	82.6 ± 12.7	76.8 ± 8.0	76.8 ± 10.9	0.108
Peripheral office DBP Z-score	2.62 ± 1.57	1.47 ± 1.01	1.82 ± 1.78	0.075
Central office SBP [mmHg]	114.8 ± 9.8	109.9 ± 7.6	110.0 ± 9.9	0.117
Central office DBP [mmHg]	84.4 ± 12.9	78.4 ± 8.2	78.6 ± 10.9	0.112
Central office MAP [mmHg]	98.7 ± 11.3	93.2 ± 8.0	94.4 ± 9.4	0.121
24 h ambulatory SBP [mmHg]	132.6 ± 7.0	133.9 ± 7.0	133.6 ± 6.2	0.678
24 h ambulatory DBP [mmHg]	72.3 ± 6.5	71.7 ± 6.1	74.1 ± 7.2	0.335
24 h ambulatory MAP [mmHg]	5.1 ± 0.7	5.5 ± 1.1	5.1 ± 0.7	0.510
24 h ambulatory MAP Z-score	−0.2 ± 0.9	0.1 ± 1.2	−0.2 ± 0.9	0.253
24 h ambulatory HR [bpm]	81.3 ± 10.9	76.2 ± 10.9	80.5 ± 11.5	0.112
ECHO LVMI [g/m^2.7^]	44.5 ± 12.5	35.6 ± 10.1	37.9 ± 7.7	0.049 ^2^
aPWV [m/s]	5.2 ± 0.7	5.6 ± 1.1	5.3 ± 0.9	0.213
aPWV Z-score	−0.18 ± 0.92	0.17 ± 1.21	0.01 ± 1.23	0.527
AIxHR75 [%]	−4.1 ± 13.7	−5.1 ± 12.7	3.1 ± 12.1	0.027 ^1^
cIMT [mm]	0.44 ± 0.07	0.46 ± 0.08	0.46 ± 0.05	0.484
cIMT Z-score	1.00 ± 1.36	1.37 ± 1.57	1.64 ± 1.24	0.357

BMI—body mass index, eGFR—estimated glomerular filtration rate according to Schwartz formula, LDL—low-density lipoprotein, HDL—high-density lipoprotein, SBP—systolic blood pressure, DBP—diastolic blood pressure, MAP—mean arterial pressure, HR—heart rate, LVMI—left ventricular mass index, aPWV—aortic pulse wave velocity, AIx75HR—augmentation index normalized to heart rate 75 beats per minute, cIMT—common carotid artery intima-media thickness. ^1^—non-dippers vs. extreme dippers; ^2^—non-dippers vs. dippers; *—26/112 patients = 13 non-dippers, 7 dippers, 6 extreme-dippers.

**Table 8:** Systolic and diastolic blood pressure dipping (continuous variable) and risk for hypertension-mediated organ damage—analysis by logistic regression (unadjusted odd ratios).

HMOD	BP Dipping	*p*	Odds Ratio	95% Confidence Interval
Left ventricular hypertrophy	SBP DIP	0.061	0.896	0.799–1.005
Left ventricular hypertrophy	DBP DIP	0.326	0.966	0.900–1.036
Abnormal aPWV	SBP DIP	0.894	1.011	0.858–1.191
Abnormal aPWV	DBP DIP	0.622	1.027	0.924–1.141
Abnormal AIx75HR	SBP DIP	0.034	1.122	1.009–1.249
Abnormal AIx75HR	DBP DIP	0.015	1.095	1.017–1.177
Abnormal cIMT	SBP DIP	0.150	1.071	0.976–1.175
Abnormal cIMT	DBP DIP	0.359	1.029	0.968–1.092
Abnormal UAE	SBP DIP	0.304	0.956	0.878–1.041
Abnormal UAE	DBP DIP	0.448	0.978	0.922–1.037
Any HMOD	SBP DIP	0.408	1.028	0.963–1.097
Any HMOD	DBP DIP	0.258	1.027	0.981–1.075

HMOD—hypertension-mediated organ damage, BP—blood pressure, SBP—systolic blood pressure, DIP—blood pressure dipping, DBP—diastolic blood pressure, aPWV—aortic pulse wave velocity, AIx75HR—augmentation index normalized to heart rate 75 beats per minute, cIMT—common carotid artery intima-media thickness, UAE—urinary albumin excretion.

## Data Availability

The data presented in this study are available on request from the corresponding author.
